# Supplementary material for: Do Molecular Fingerprints Identify Diverse Active Drugs in Large-Scale Virtual Screening? (No)
Source: Pharmaceuticals (Basel). 2024 Jul 26;17(8):992. doi: 10.3390/ph17080992 (PMC11356940; doi:10.3390/ph17080992)
Supplement: Supplementary file 1 [file pharmaceuticals-17-00992-s001.zip › pharmaceuticals-3074092-supplementary.pdf]

Supplementary Material for

Do Molecular Fingerprints Identify Diverse Active Drugs in Large-Scale Virtual Screening? (No)

Vishwesh Venkatraman [1,*] , Jeremiah Gaiser [2], Daphne Demekas [3] , Amitava Roy [4,5] , Rui Xiong [6]

and Travis J. Wheeler [3,*]

[1] *Department of Chemistry, Norwegian University of Science and Technology, 7034 Trondheim, Norway*
[2] *School of Information, University of Arizona, Tucson, AZ 85721, USA*
[3] *R. Ken Coit College Pharmacy, University of Arizona, Tucson, AZ 85721, USA*
[4] *Rocky Mountain Laboratories, Bioinformatics and Computational Biosciences Branch, Office of Cyber Infrastructure and Computational Biology, National Institute of Allergy and Infectious Diseases, National Institutes of Health, Hamilton, MT 59840, USA*
[5] *Department of Biomedical and Pharmaceutical Sciences, University of Montana, Missoula, MT 59812, USA*
[6] *Department of Pharmacology & Toxicology, University of Arizona, Tucson, AZ 85721, USA*

Since, the ROC AUC is not well-suited for evaluating the early recognition of active molecules, metrics such as the Boltzmann-Enhanced Discrimination of the Receiver Operating Characteristic[1] (BEDROC) and the sum of the log of ranks statistic[2, 3] (SLR) have been proposed. To capture early enrichment, we have calculated the Boltzmann-Enhanced Discrimination of the Receiver Operating Characteristic (BEDROC) metric which assigns more weight to early ranked molecules:

$$BEDROC = \frac{\sum_{i=1}^{n} e^{-\alpha r_i/N}}{R_a\left(\frac{1-e^{-\alpha}}{e^{\alpha/N}-1}\right)} \times \frac{R_a \sinh(\alpha/2)}{\cosh(\alpha/2) - \cosh(\alpha/2 - \alpha R_a)} + \frac{1}{1-e^{\alpha(1-R_a)}} \tag{S1}$$

where n is the number of actives, N the total number of compounds, $R_a = n/N$, the ratio of actives to inactives in the dataset and r_i, the rank of the ith active. The parameter α is used to emphasize early recognition. The value of α value is chosen so that 0.5% ($\alpha = 321.9$), 2% ($\alpha = 80.5$) or 8% ($\alpha = 20.0$) of the top-ranked molecules account for 80% of the BEDROC score.

We also report the normalized form of the sum of log rank statistic[3] (NSLR):

$$NSLR = \frac{-\sum_{i=1}^{n} \log \frac{r_i}{N}}{-\sum_{i=1}^{n} \log \frac{i}{N}} \tag{S2}$$

where n is the number of actives among the N available compounds and r_i is the rank of the ith active. The negative logarithm emphasizes early recognition. The denominator in the equation provides a theoretical maximum when a VS method ranks all actives within the first n positions. NSLR varies between 0 and 1, where the latter is the best achievable ranking.

FP	DEKOIS	DUDE	MUV	LIT-PCBA	DEKOIS	DUDE	MUV	LIT-PCBA
		**AUC_S**				**AUC_L**		
AVALON	0.72	0.73	0.60	0.55	0.70	0.77	0.60	0.55
ECFP0	0.70	0.77	0.53	0.50	0.70	0.77	0.53	0.50
ECFP2	0.77	0.81	0.54	0.51	0.77	0.81	0.54	0.51
ECFP4	0.76	0.80	0.54	0.51	0.77	0.81	0.54	0.51
ECFP6	0.75	0.78	0.54	0.52	0.77	0.80	0.54	0.51
FCFP0	0.66	0.69	0.54	0.52	0.66	0.69	0.54	0.52
FCFP2	0.76	0.75	0.55	0.52	0.76	0.75	0.55	0.51
FCFP4	0.78	0.76	0.54	0.52	0.78	0.76	0.55	0.51
FCFP6	0.78	0.75	0.54	0.52	0.78	0.76	0.54	0.51
		**DRF_S**				**DRF_L**		
AVALON	0.30	0.18	0.85	0.97	0.25	0.16	0.89	0.98
ECFP0	0.33	0.13	0.97	0.96	0.33	0.13	0.97	0.96
ECFP2	0.19	0.09	0.99	1.01	0.18	0.08	0.96	0.98
ECFP4	0.19	0.09	0.99	1.00	0.18	0.09	0.99	0.99
ECFP6	0.20	0.10	0.99	0.98	0.19	0.09	0.99	1.01
FCFP0	0.35	0.23	0.41	0.42	0.35	0.23	0.41	0.42
FCFP2	0.24	0.19	0.93	1.01	0.24	0.19	0.93	1.00
FCFP4	0.20	0.15	0.93	1.01	0.20	0.16	0.93	1.01
FCFP6	0.20	0.15	0.96	0.98	0.20	0.16	0.94	1.02

Table S1: Summary of the VS screening performances using extended length fingerprints. The impact of fingerprint length on the VS metrics was assessed using the default ("_S") length (1024 bits) and the long ("_L") form (16384 bits).

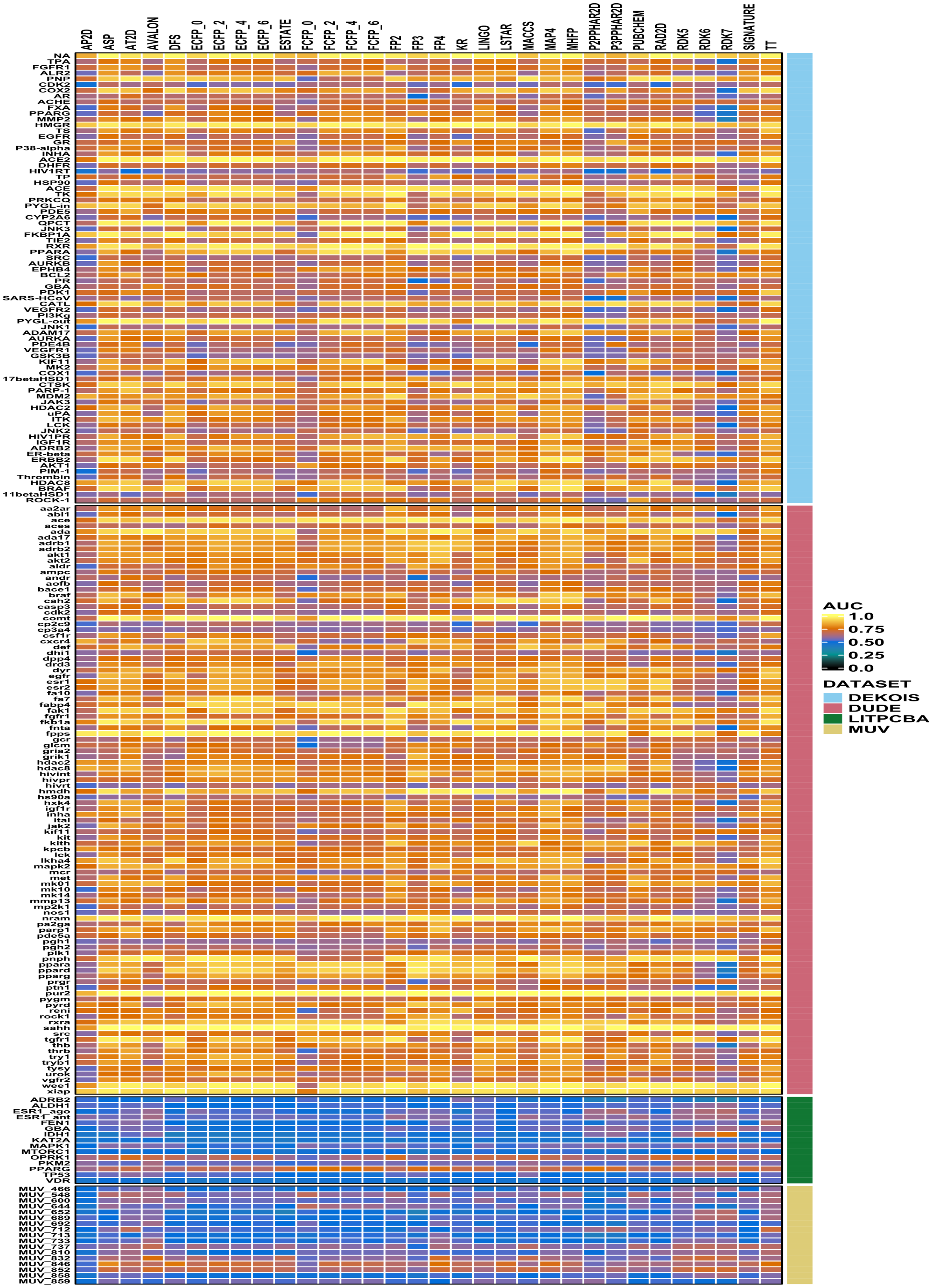

Figure S1: Heatmap of the area under the curve (AUC) obtained by the different fingerprints for the targets in the benchmark datasets.

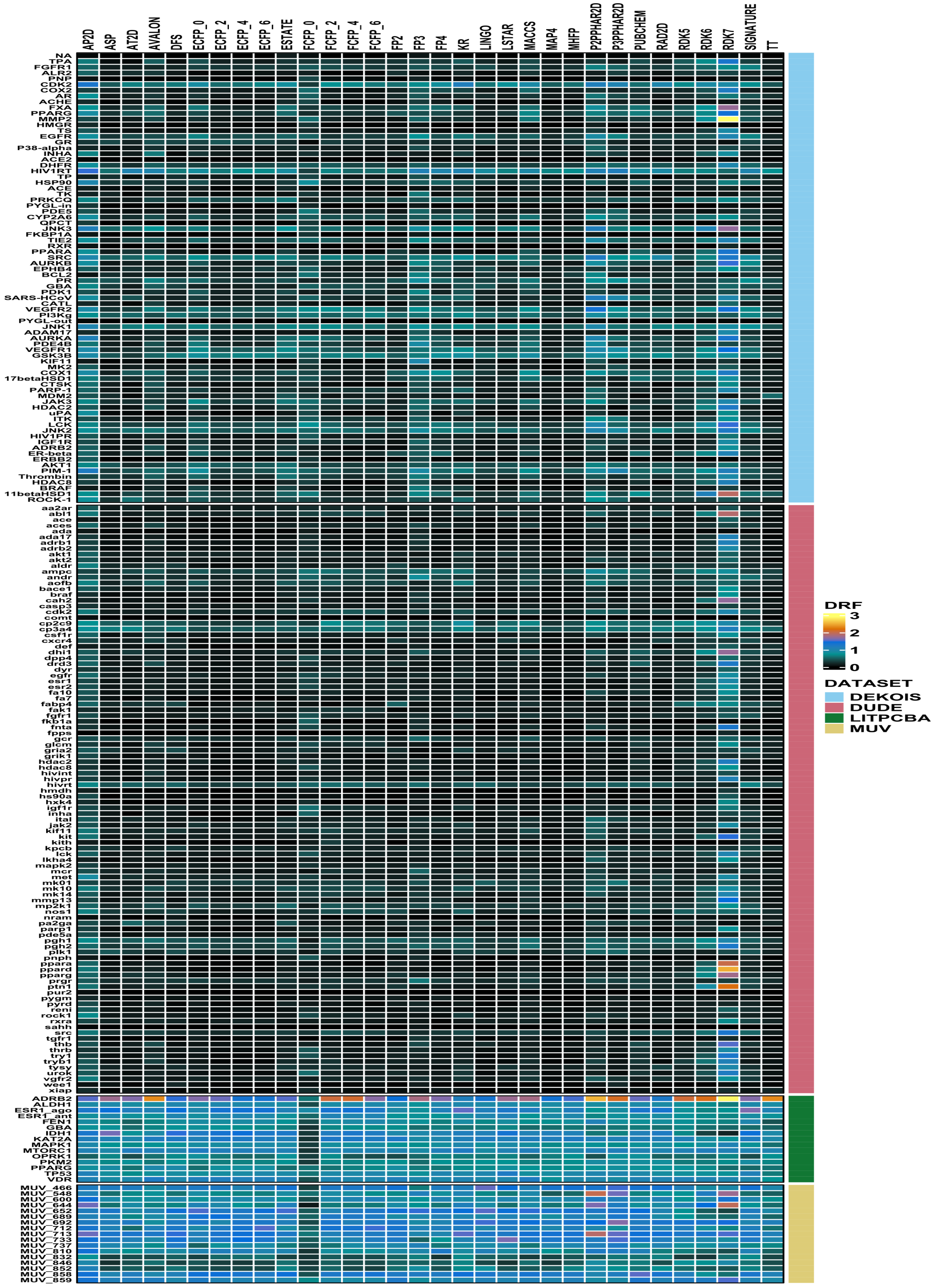

Figure S2: Heatmap of the decoy retention factor ($DRF_{0.1}$) obtained by the different fingerprints for the targets in the benchmark datasets.

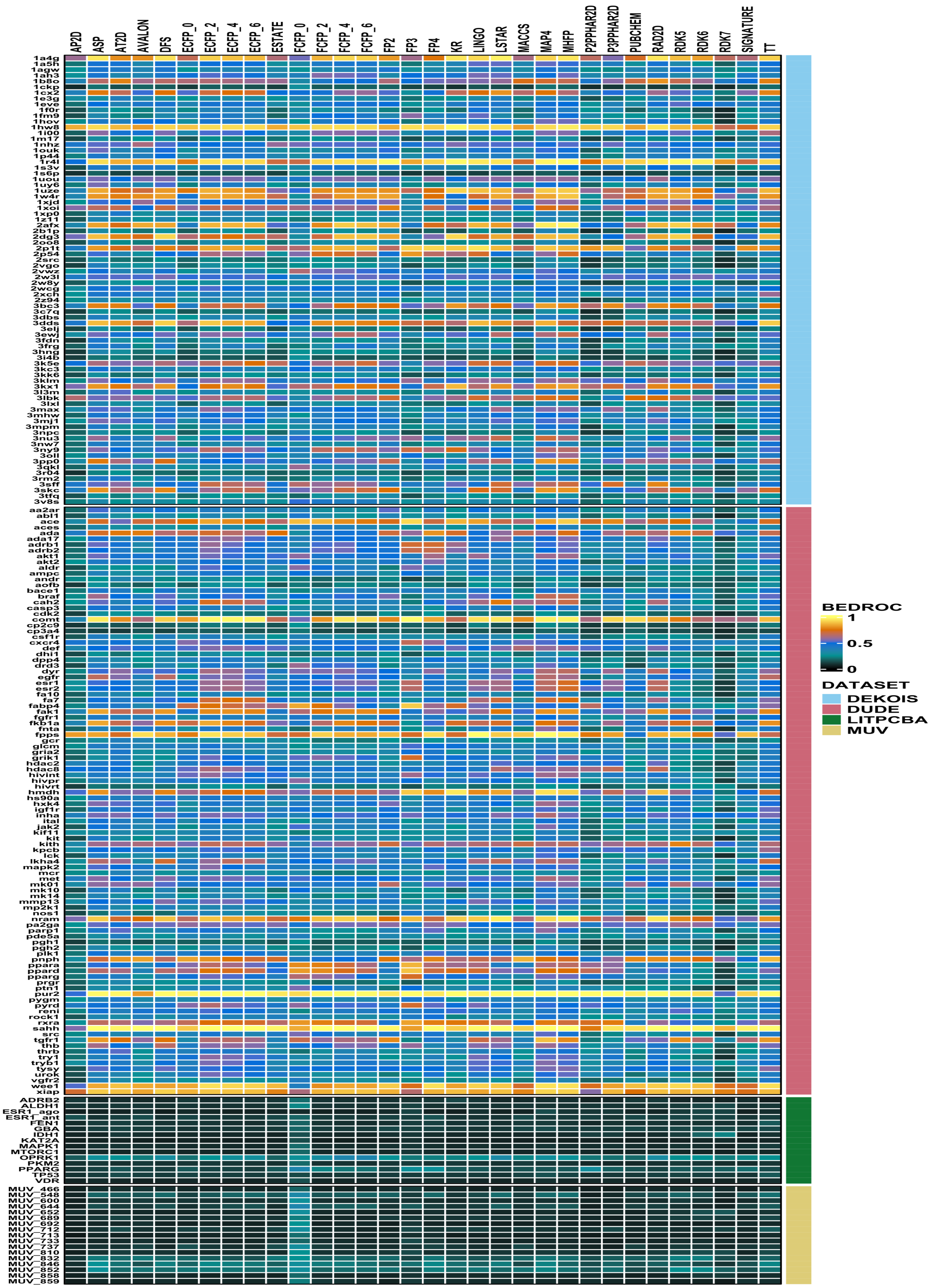

Figure S3: Heatmap of the BEDROC ($\alpha = 20$) values obtained by the different fingerprints for the targets in the benchmark datasets.

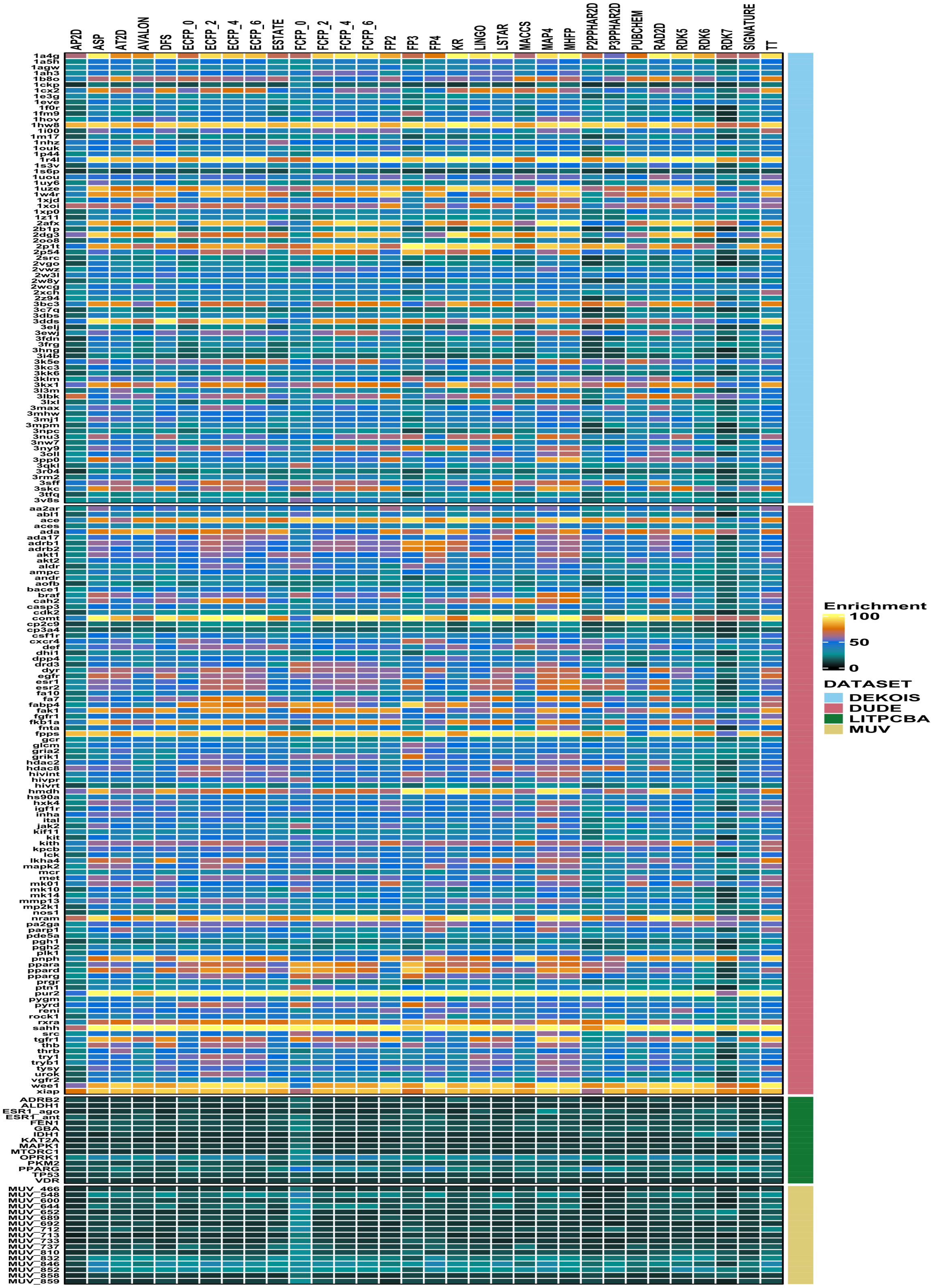

Figure S4: Heatmap of the enrichment factor (top 8%) obtained by the different fingerprints for the targets in the benchmark datasets.

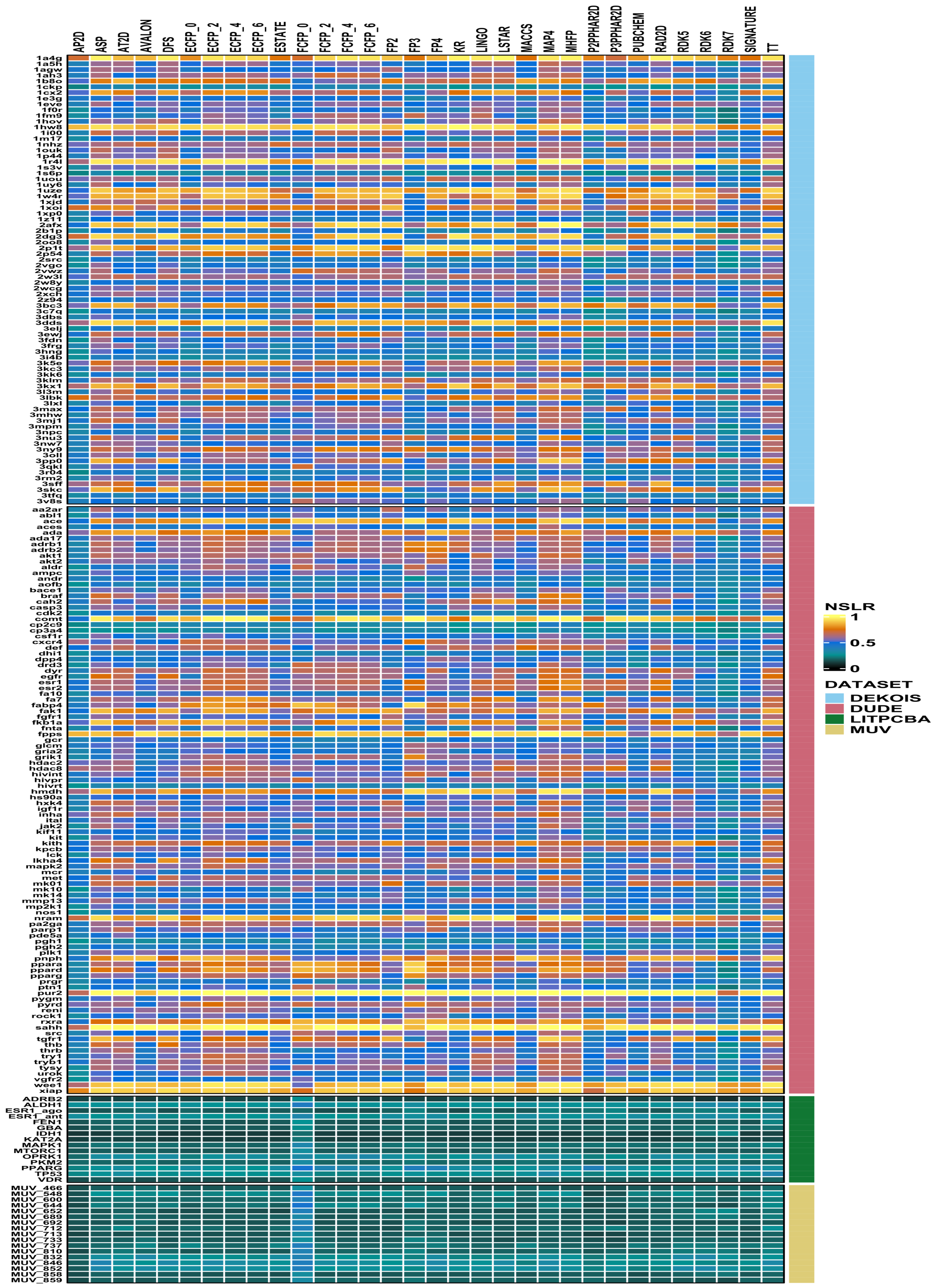

Figure S5: Heatmap of the NSLR values obtained by the different fingerprints for the targets in the benchmark datasets.

PPARG

Active Query

CN(CCOc1ccc(CC(C([N-]2)=O)SC2=O)cc1)c1ncccc1

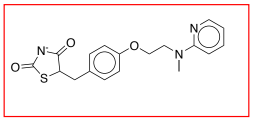

Active hit with Tanimoto >=0.5 (ECFP4)

CCc1cnc(CCOc2ccc(CC(C([N-]3)=O)SC3=O)cc2)cc1

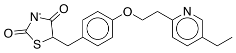

Same scaffold; non-obvious

Figure S6: In the LIT-PCBA analysis, early enrichment was observed for PPARG – for all Tanimoto coefficients $t > 0.2$, the fraction of actives with Tanimoto score $> t$ is much larger than the fraction of decoys with that score (see Figure 2 in the main text). We manually inspected the structure of the 1 compound with Tanimoto score > 0.5 to the initial query. This compound is built on the same scaffold as the query (in red), but is a non-obvious variant on that scaffold.

GBA

Active Query

Cc(cc1)cc(Cl)c1NC(CN(c1cc(C)cc(C)c1)S(c(c(C)nc([O-])n1)c1[O-])(=O)=O)=O

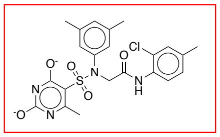

Active hits with Tanimoto >=0.5 (ECFP4)

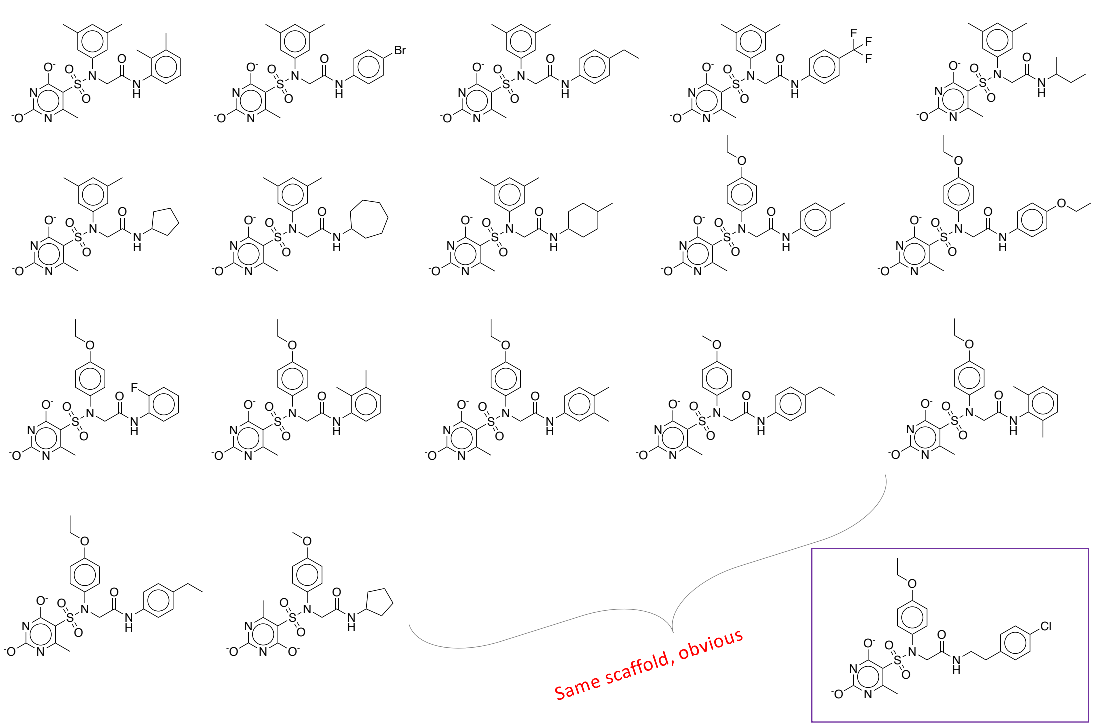

Same scaffold; non-obvious.

Cc1cc(N(CC(Nc2c(C)c(C)ccc2)=O)S(c(c(C)nc([O-])n2)c2[O-])(=O)=O)cc(C)c1
Cc1cc(N(CC(Nc(cc2)ccc2Br)=O)S(c(c(C)nc([O-])n2)c2[O-])(=O)=O)cc(C)c1
CCc(cc1)ccc1NC(CN(c1cc(C)cc(C)c1)S(c(c(C)nc([O-])n1)c1[O-])(=O)=O)=O
Cc1cc(N(CC(Nc2ccc(C(F)(F)F)cc2)=O)S(c(c(C)nc([O-])n2)c2[O-])(=O)=O)cc(C)c1
CCC(C)NC(CN(c1cc(C)cc(C)c1)S(c(c(C)nc([O-])n1)c1[O-])(=O)=O)=O
Cc1cc(N(CC(NC2CCCC2)=O)S(c(c(C)nc([O-])n2)c2[O-])(=O)=O)cc(C)c1
Cc1cc(N(CC(NC2CCCCCC2)=O)S(c(c(C)nc([O-])n2)c2[O-])(=O)=O)cc(C)c1
CC(CC1)CCC1NC(CN(c1cc(C)cc(C)c1)S(c(c(C)nc([O-])n1)c1[O-])(=O)=O)=O
CCOc(cc1)ccc1N(CC(Nc1ccc(C)cc1)=O)S(c(c(C)nc([O-])n1)c1[O-])(=O)=O
CCOc(cc1)ccc1NC(CN(c(cc1)ccc1OCC)S(c(c(C)nc([O-])n1)c1[O-])(=O)=O)=O
CCOc(cc1)ccc1N(CC(Nc(cccc1)c1F)=O)S(c(c(C)nc([O-])n1)c1[O-])(=O)=O
CCOc(cc1)ccc1N(CC(Nc1c(C)c(C)ccc1)=O)S(c(c(C)nc([O-])n1)c1[O-])(=O)=O
CCOc(cc1)ccc1N(CC(Nc1cc(C)c(C)cc1)=O)S(c(c(C)nc([O-])n1)c1[O-])(=O)=O
CCc(cc1)ccc1NC(CN(c(cc1)ccc1OC)S(c(c(C)nc([O-])n1)c1[O-])(=O)=O)=O
CCOc(cc1)ccc1N(CC(Nc1c(C)cccc1C)=O)S(c(c(C)nc([O-])n1)c1[O-])(=O)=O
CCc(cc1)ccc1NC(CN(c(cc1)ccc1OCC)S(c(c(C)nc([O-])n1)c1[O-])(=O)=O)=O
Cc1nc([O-])nc([O-])c1S(N(CC(NC1CCCC1)=O)c(cc1)ccc1OC)(=O)=O
CCOc(cc1)ccc1N(CC(NCCc(cc1)ccc1Cl)=O)S(c(c(C)nc([O-])n1)c1[O-])(=O)=O

Figure S7: In the LIT-PCBA analysis, early enrichment was observed for GBA – for all Tanimoto coefficients $t > 0.2$, the fraction of actives with Tanimoto score $> t$ is much larger than the fraction of decoys with that score (see Figure 2). There are 163 actives in the GBA data set. We manually inspected the structures of the 18 compounds with Tanimoto score > 0.5 to the initial query. All 18 appear are built on the same scaffold as the query (in red), and all but one is an obvious variation that should be identified through standard enumeration (i.e. no new scaffolds are explored).

References

[1] Jean-François Truchon and Christopher I. Bayly. Evaluating virtual screening methods: good and bad metrics for the early recognition problem. *Journal of Chemical Information and Modeling*, 47(2):488–508, 2007.

[2] Wei Zhao, Kirk E Hevener, Stephen W White, Richard E Lee, and James M Boyett. A statistical framework to evaluate virtual screening. *BMC Bioinformatics*, 10(1), 2009.

[3] Vishwesh Venkatraman, Violeta I. Pérez-Nueno, Lazaros Mavridis, and David W. Ritchie. Comprehensive comparison of ligand-based virtual screening tools against the DUD data set reveals limitations of current 3d methods. *Journal of Chemical Information and Modeling*, 50(12):2079–2093, 2010.
